# CNNM2 Mutations Cause Impaired Brain Development and Seizures in Patients with Hypomagnesemia

**DOI:** 10.1371/journal.pgen.1004267

**Published:** 2014-04-03

**Authors:** Francisco J. Arjona, Jeroen H. F. de Baaij, Karl P. Schlingmann, Anke L. L. Lameris, Erwin van Wijk, Gert Flik, Sabrina Regele, G. Christoph Korenke, Birgit Neophytou, Stephan Rust, Nadine Reintjes, Martin Konrad, René J. M. Bindels, Joost G. J. Hoenderop

**Affiliations:** 1 Department of Physiology, Radboud Institute for Molecular Life Sciences, Radboud university medical center, Nijmegen, The Netherlands; 2 Department of General Pediatrics, University Children's Hospital, Münster, Germany; 3 Department of Otorhinolaryngology, Radboud university medical center, Nijmegen, The Netherlands; 4 Department of Organismal Animal Physiology, Institute for Water and Wetland Research, Radboud University Nijmegen, Nijmegen, The Netherlands; 5 Department of Neuropediatrics, Children's Hospital, Oldenburg, Germany; 6 Department of Neuropediatrics, St. Anna Children's Hospital, Medical University Vienna, Vienna, Austria; 7 Leibniz Institute of Arteriosclerosis Research, University of Münster, Münster, Germany; 8 Institute of Human Genetics, University of Cologne, Cologne, Germany; Columbia University, United States of America

## Abstract

Intellectual disability and seizures are frequently associated with hypomagnesemia and have an important genetic component. However, to find the genetic origin of intellectual disability and seizures often remains challenging because of considerable genetic heterogeneity and clinical variability. In this study, we have identified new mutations in *CNNM2* in five families suffering from mental retardation, seizures, and hypomagnesemia. For the first time, a recessive mode of inheritance of *CNNM2* mutations was observed. Importantly, patients with recessive *CNNM2* mutations suffer from brain malformations and severe intellectual disability. Additionally, three patients with moderate mental disability were shown to carry *de novo* heterozygous missense mutations in the *CNNM2* gene. To elucidate the physiological role of CNNM2 and explain the pathomechanisms of disease, we studied CNNM2 function combining *in vitro* activity assays and the zebrafish knockdown model system. Using stable Mg^2+^ isotopes, we demonstrated that CNNM2 increases cellular Mg^2+^ uptake in HEK293 cells and that this process occurs through regulation of the Mg^2+^-permeable cation channel TRPM7. In contrast, cells expressing mutated CNNM2 proteins did not show increased Mg^2+^ uptake. Knockdown of *cnnm2* isoforms in zebrafish resulted in disturbed brain development including neurodevelopmental impairments such as increased embryonic spontaneous contractions and weak touch-evoked escape behaviour, and reduced body Mg content, indicative of impaired renal Mg^2+^ absorption. These phenotypes were rescued by injection of mammalian wild-type *Cnnm2* cRNA, whereas mammalian mutant *Cnnm2* cRNA did not improve the zebrafish knockdown phenotypes. We therefore concluded that CNNM2 is fundamental for brain development, neurological functioning and Mg^2+^ homeostasis. By establishing the loss-of-function zebrafish model for CNNM2 genetic disease, we provide a unique system for testing therapeutic drugs targeting CNNM2 and for monitoring their effects on the brain and kidney phenotype.

## Author Summary

Mental retardation affects 1–3% of the population and has a strong genetic etiology. Consequently, early identification of the genetic causes of mental retardation is of significant importance in the diagnosis of the disease, as predictor of the progress of the disease and for the determination of treatment. In this study, we identify mutations in the gene encoding for cyclin M2 (CNNM2) to be causative for mental retardation and seizures in patients with hypomagnesemia. Particularly, in patients with a recessive mode of inheritance, the intellectual disability caused by dysfunctional CNNM2 is dramatically severe and is accompanied by severely limited motor skills and brain malformations suggestive of impaired early brain development. Although hypomagnesemia has been associated to several neurological diseases, Mg^2+^ status is not regularly assessed in patients with seizures and mental disability. Our findings establish CNNM2 as an important protein for renal magnesium handling, brain development and neurological functioning, thus explaining the physiology of human disease caused by (dysfunctional) mutations in CNNM2. CNNM2 mutations should be taken into account in patients with seizures and mental disability, specifically in combination with hypomagnesemia.

## Introduction

Brain defects including seizures, migraine, depression and intellectual disability are frequently associated with hypomagnesemia [1]. Indeed, low Mg^2+^ concentrations may cause epileptiform activity during development [2]. Specifically, the Mg^2+^ channel transient receptor potential melastatin 7 (TRPM7) is essential for brain function and development [3]. Interestingly, patients with genetic defects in TRPM6, a close homologue of TRPM7, may have neurological complications [4]. Although TRPM6 and TRPM7 share similar Mg^2+^ transporting properties, they are differentially expressed and regulated [5]. TRPM7 is a ubiquitously expressed protein regulating intracellular Mg^2+^ levels in a broad range of cells, whereas TRPM6 is localized in the luminal membrane of renal and intestinal epithelia involved in Mg^2+^ absorption [1], [6]–[8]. Recently, we have identified mutations in the gene encoding cyclin M2 (CNNM2) in two unrelated families with dominant isolated hypomagnesemia (CNNM2 [MIM 607803]) [9]. Patients suffered from symptoms associated with low serum Mg^2+^ levels (0.3–0.5 mM) such as tremors, headaches and muscle weakness. The role of CNNM2 in the kidney for the maintenance of serum Mg^2+^ levels can be traced to the distal convoluted tubule (DCT), where also TRPM6 is expressed. Here, CNNM2 is present in the basolateral membrane of DCT cells and its expression is regulated by dietary Mg^2+^ availability [9]–[10].

Although CNNM2 has been proposed as a Mg^2+^ transporter in overexpression studies in *Xenopus* oocytes [11], Mg^2+^ transport could not be directly measured in mammalian cells using patch clamp analysis [9]. On the other hand, modelling of the CNNM2 cystathionine β-synthase (CBS) domain resulted in the identification of a Mg^2+^-ATP binding site, suggesting a role in Mg^2+^ sensing within the cell [12]. Consequently, the molecular mechanism explaining the role of CNNM2 in DCT-mediated Mg^2+^ transport remains to be elucidated.

The *CNNM2* gene is ubiquitously expressed in mammalian tissues, most prominently in kidney, brain and lung [12]–[13]. Although the role of CNNM2 beyond the kidney has never been studied, genome wide association studies have related the *CNNM2* locus to blood pressure, coronary artery disease and schizophrenia, suggesting an important role of CNNM2 in the cardiovascular system and brain [14]–[15]. CNNM2 is widely conserved among species. In zebrafish (*Danio rerio*), a frequently used model for ion homeostasis and human genetic diseases in general [16]–[17], the *cnnm2* gene is duplicated and two paralogues, *cnnm2a* and *cnnm2b*, are described [18]. Both paralogues share a high conservation with human *CNNM2* (79% amino acid identity). In detail, transcripts are abundantly expressed in zebrafish brain and in ionoregulatory organs such as kidney and gills, which act as a pseudokidney in fish [18]. Consistent with the regulation of *CNNM2* transcripts in mammals [11], the expression of *cnnm2a* and *cnnm2b* is regulated by Mg^2+^ *in vivo* [18].

In the present study, we aim to elucidate the function of CNNM2 in brain and kidney. Hence, we can demonstrate the genetic origin of symptoms in five unrelated families suffering from a distinct phenotype of mental retardation, seizures and hypomagnesemia, where we have identified novel mutations in the *CNNM2* gene. By combining functional analyses and a loss-of-function approach in the zebrafish model, we provide functional evidence for a key role of CNNM2 in brain development, neurological activity and renal Mg^2+^ handling.

## Results

### Patients

**Figure 1:**
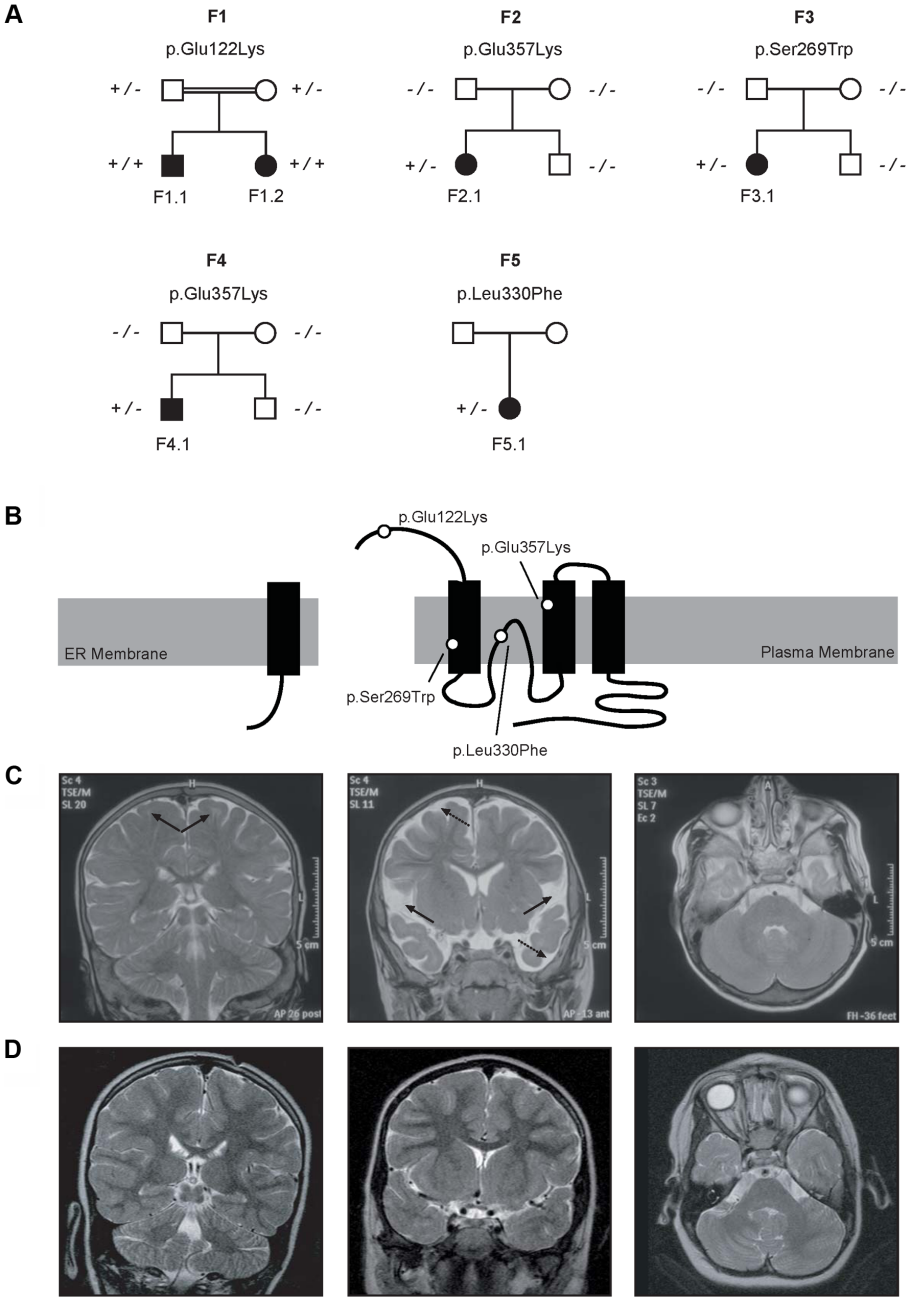
Pedigrees and magnetic resonance imaging (MRI) studies of families with primary hypomagnesemia. (A) Pedigrees of families F1–F5. Filled symbols represent affected individuals, mutant alleles are indicated by a minus (−) and plus (+) sign, respectively. (B) Localization of the mutations in the CNNM2 protein structure (Uniprot Q9H8M5). CNNM2 contains a long signal peptide (64 amino acids) that is cleaved at the membrane of the endoplasmic reticulum. The remaining part of the CNNM2 protein is trafficked to the plasma membrane, where it becomes functionally active. White dots show the locations of the mutations. (C–D) MRI of the brain of patient F1.1 (C, T2 weighed images) and patient F2.1 (D, T2 weighed images). Left: Coronal images demonstrating a defect in myelinization of U-fibers (arrows) in patient F1.1 in contrast to a normal myelin pattern in patient F2.1. Center: Coronal T2 weighted images showing widened outer cerebrospinal liquor spaces (dashed arrows) and lack of opercularization (solid arrows) in patient F1.1, whereas a regular brain volume and insular lobe is observed in patient F2.1. Right: Absence of cerebellar structural abnormalities in patients F1.1 and F2.1 on axial T2 weighted images at the level of the trigeminal nerve.

Patients F1.1 and F1.2 presented in the neonatal period with cerebral convulsions. Serum Mg^2+^ levels at manifestation were found to be 0.5 mM in both patients (Table 1). Convulsions were refractory to conventional antiepileptic medications. Intravenous Mg^2+^ supplementation with ∼1 mmol/kg body weight/day was initiated after oral Mg^2+^ failed to correct serum Mg^2+^ levels. However, seizure activity continued even in face of normomagnesemia. An extensive analysis for infectious causes or inborn errors of metabolism did not yield any positive results. Ultrasound examination of the kidneys did not reveal nephrocalcinosis, whereas basal ganglia calcifications were noted in early central nervous system (CNS) sonographies. During follow-up, severe developmental delay was noted accompanied by microcephaly (head circumference below third percentile for age and sex in both patients). A magnetic resonance imaging (MRI) at 5.5 years of age in patient F1.1 showed wide supratentorial outer cerebrospinal liquor spaces with failure of opercularization together with a significantly reduced myelinization of the white matter tract (Figure 1C–D). The severe degree of intellectual disability, which became apparent with increasing age comprised major deficits in cognitive function, the inability to verbally communicate, and severely limited motor skills. Both children are not able to perform main activities of daily living and require full-time care by an attendant. Seizure activity is sufficiently controlled in the older brother by valproate and lamotrigine, electroencephalography (EEGs) merely shows generalized slowing, but no epileptic activity. In contrast, the younger sister suffers from ongoing generalized, myoclonic seizures despite antiepileptic treatment with valproate and levetiracetame. Laboratory investigations during follow-up demonstrated persistent hypomagnesemia of ∼0.6 mM despite oral Mg^2+^ supplementation.

**Table 1:** Clinical and biochemical data of patients.

Patient	F1.1	F1.2	F2.1	F3.1	F4.1	F5.1
**Gender**	Male	Female	Female	Female	Male	Female
**Ethnicity**	Serbian	Serbian	German	German	German	Polish
**Age at manifestation**	1 day	6 days	7 months	1 years	4 months	16 years
**Follow-up**	12 years	8 years	12 years	20 years	12 years	None
**Symptoms at manifestation**	Seizures	Seizures	Seizures	Seizures, Paresthesia	Seizures	Myoclonus, Paresthesia
**Treatment**	Valproate, Lamotrigine	Valproate, Levetiracetame	Phenobarbital	Valproate	Clobazam	Unknown
**Neuroimaging**	Myelinization defects, Opercularization defect, Widened outer cerebrospinal liquor spaces		Normal	Normal	Normal	Unknown
**Mental retardation**	Severe	Severe	Moderate	Moderate	Moderate	Mild
**Cognitive function**			IQ 55–57	IQ 55–59	Autism	Unknown
**Speech/Communication**	No verbal speech, Limited communication skills	No verbal speech, Limited communication skills	Expressive language disorder	Expressive language disorder	Limited speech and vocabulary	Unknown
**Additional symptoms**	Very limited motor skills	Very limited motor skills	Impaired motor skills, severe obesity	Impaired motor skills, severe obesity	Impaired motor skills	Unknown
**Initial serum Mg^2+^ (mmol/L)**	0.5	0.5	0.56	0.44	0.5	0.66
**Follow-up serum Mg^2+^ (mmol/L)**	0.66	0.54	0.56	0.53	0.68	-
**Mutation (DNA level)**	c.364G>A	c.364G>A	c.1069G>A	c.806C>G	c.1069G>A	c.988C>T
**Mutation (Protein level)**	p.Glu122Lys	p.Glu122Lys	p.Glu357Lys	p.Ser269Trp	p.Glu357Lys	p.Leu330Phe
**Zygosity**	Homozygous	Homozygous	Heterozygous	Heterozygous	Heterozygous	Heterozygous

Patients F2.1, F3.1, and F4.1 presented with seizures during infancy (between 4 and 12 months of age). Laboratory evaluation yielded isolated hypomagnesemia of ∼0.5 mM (Table 1). Urine analyses demonstrated inappropriate fractional excretion for Mg^2+^ in face of persistent hypomagnesemia. In addition, the renal Mg^2+^ leak was verified by Mg^2+^ loading tests in patients F2.1 and F4.1 as described before [19]. Urinary calcium excretion rates were normal, renal ultrasound excluded the presence of nephrocalcinosis. After acute therapy with intravenous Mg^2+^, the patients received a continuous oral Mg^2+^ supplementation of 0.5 to 1 mmol/kg body weight/day of elemental Mg^2+^. This oral therapy however failed to correct the hypomagnesemia, serum Mg^2+^ remained in the subnormal range in all three children. Because of recurrent cerebral seizures, patients F2.1 to F4.1 received diverse antiepileptic medications. Currently only patient F4.1 is still treated with clobazam.

In all three patients (F2.1 to F4.1), a significant degree of intellectual disability was already noted in early childhood with delayed speech development, but also impaired motor as well as cognitive skills. In addition, patient F4.1 was noted to exhibit disturbed social interaction, abnormal verbal and non-verbal communication, as well as stereotyped behaviour and finally received the formal diagnosis of early onset autism. All three patients F2.1 to F4.1 were not able to attend regular schools. Standardized intelligence testing in patients F2.1 and F3.1 revealed a significant degree of mental retardation (see Table 1). While patients F2.1 and F3.1 are currently living with their parents, patient F4.1 is placed in a home for children with mental illness because of episodes of violence and destructive behaviour. The parents of all three children (F2.1–F4.1) had normal serum Mg^2+^ levels and no signs of intellectual disability.

Finally, patient F5.1 presented with muscle spasms and dysesthesia in adolescence. Serum Mg^2+^ levels were found to be low (∼0.6 mM). Because of concomitant borderline hypokalemia, she was suspected to have Gitelman syndrome ([MIM 263800]) and received oral Mg^2+^ and K^+^ supplements. Also this patient exhibited a mild degree of intellectual disability. Unfortunately, she was not available for further examination.

### CNNM2 mutations in patients with mental retardation

Common genetic causes of mental retardation were excluded in patients F1.1 and F2.1 by array CGH (comparative genomic hybridization). The presence of two affected siblings together with the suspected parental consanguinity in family F1 suggested an autosomal-recessive pattern of inheritance. Therefore, we subjected patients F1.1 and F1.2 to homozygosity mapping which, at a cut-off size of >1.7 megabases (Mb), yielded eleven critical intervals on autosomes with a cumulative size of 62 Mb. The gene list generated from these loci included 322 RefSeq genes and putative transcripts. *CNNM2* in a critical interval of 7.1 Mb on chromosome 10 emerged as the most promising candidate gene because of its known role in Mg^2+^ metabolism [9], [11]–[12], [20]. Conventional Sanger sequencing of the complete coding region of the *CNNM2* gene revealed a homozygous mutation, c.364G>A, leading to a non-conservative amino acid substitution of glutamate to lysine at position 122 of the CNNM2 protein (p.Glu122Lys, Figure 1A–B). The mutation was present in heterozygous state in both parents. After discovery of this homozygous mutation in patients F1.1 and F1.2, a larger cohort (n = 34) of patients with Mg^2+^ deficiency of unknown origin was screened for mutations in the *CNNM2* gene. Mutations in heterozygous state were discovered in patients F2.1 to F5.1 (Table 1). However, sequencing of the complete coding region and adjacent exon-intron boundaries did not reveal a second pathogenic allele. Next, we examined the *CNNM2* gene in parents and unaffected siblings of families F2 to F4. The mutations previously identified in our patients were not detected in either of the parents pointing to *de novo* mutational events. Interestingly, patients F2.1 and F4.1 exhibited the same mutation, p.Glu357Lys (c.1069G>A), affecting a highly conserved amino acid residue in the 2^nd^ membrane-spanning domain of the CNNM2 protein. Also the p.Ser269Trp (c.806C>G) mutation detected in patient F3.1 affects a highly conserved residue located in the 1^st^ transmembrane domain. All three mutations were ranked “probably damaging” by Polyphen-2 when tested for functional consequences of the mutations (p.Glu122Lys, p.Ser269Trp and p.Glu357Lys with scores of 0.981, 1.000 and 1.000, respectively). Finally, in patient F5.1 with a late manifestation and putatively milder phenotype, the variant p.Leu330Phe (c.988C>T) was identified in heterozygous state. This variant affects an amino acid residue conserved among mammals, however a phenylalanine appears at this position in certain fish species. The variant is predicted to be possibly damaging by Polyphen-2 with a score of 0.711. None of the identified variants was detected in 204 controls or present in publically available exome data.

### CNNM2 increases Mg^2+^ transport

**Figure 2:**
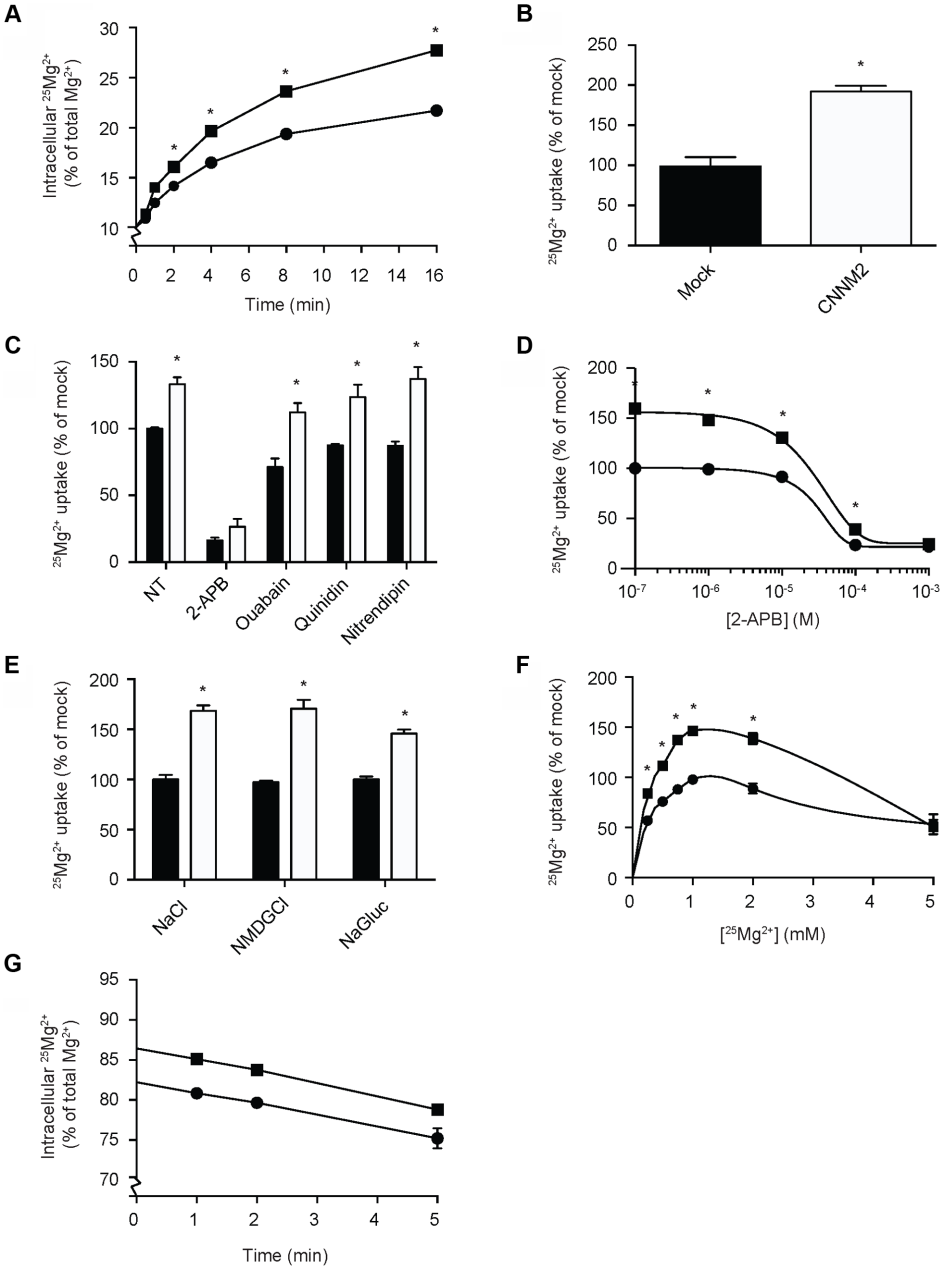
CNNM2 increases Mg^2+^ uptake in HEK293 cells. (A) Time curve of ^25^Mg^2+^ uptake in mock (circles) and CNNM2 (squares) transfected cells. (B) Representation of the normalized Mg^2+^ uptake after 5 minutes. (C) ^25^Mg^2+^ uptake in the presence of inhibitors of ion transporters, black bars represent mock cells and white bars represent CNNM2-transfected cells. (D) Dose-response curve of ^25^Mg^2+^ transport inhibition by 2-APB in mock (circles) and CNNM2 (squares) transfected cells. (E) The effect of Na^+^ and Cl^−^ availability on ^25^Mg^2+^ uptake in mock (black bars) and CNNM2 (white bars) transfected cells. (F) ^25^Mg^2+^ uptake as a function of extracellular ^25^Mg^2+^ availability in mock (circles) and CNNM2 (squares) transfected cells. (G) ^25^Mg^2+^ extrusion in mock (circles) and CNNM2 (squares) transfected cells. Each data point represent the mean of 3 independent experiments ± SEM. * indicates significant differences compared to mock (*P*<0.05).

To clarify the function of CNNM2, Human Embryonic Kidney (HEK293) cells were transiently transfected with mouse *Cnnm2* or mock constructs and examined for Mg^2+^ transport capacity using the stable ^25^Mg^2+^ isotope. At baseline, approximately 10% of the total intracellular Mg^2+^ content consists of ^25^Mg^2+^, which is the natural abundance of ^25^Mg^2+^ [21]. By incubating the cells in a physiological buffer containing pure ^25^Mg^2+^, the intracellular ^25^Mg^2+^ concentration increases over time. Interestingly, *Cnnm2* expressing cells displayed a higher ^25^Mg^2+^ uptake compared to mock cells (Figure 2A). After 5 minutes, *Cnnm2*-expressing cells had approximately 2 times more ^25^Mg^2+^ uptake than mock-transfected cells (Figure 2B). All further experiments were performed at the 5 minutes time point to cover the exponential phase of the uptake. To reduce the background in ^25^Mg^2+^ uptake, inhibitors of known Mg^2+^ channels and transporters were added during the uptake process; 2-APB to inhibit TRPM7 [22], Ouabain to block the Na^+^-K^+^-ATPase [23], Quinidin for SLC41A1 [24] and Nitrendipin [25] for silencing MagT1 activity (Figure 2C). Only 2-APB was capable of significantly inhibiting ^25^Mg^2+^ uptake in HEK293 cells. Moreover, 2-APB inhibition also abolished the CNNM2-dependent increase in ^25^Mg^2+^ uptake. Dose-response experiments confirmed that the IC_50_ of 2-APB inhibition is 22 µM (Figure 2D). CNNM2-dependent ^25^Mg^2+^ uptake was found to be independent of Na^+^ and Cl^−^ availability, when uptakes were performed in N-methyl-d-glucamine (NMDG) or Gluconate buffers (Figure 2E). Interestingly, the highest CNNM2-dependent ^25^Mg^2+^ uptake was measured between 1–2 mM, suggesting a K_m_ in the physiological range of approximately 0.5 mM (Figure 2F). At high Mg^2+^ concentrations (5 mM), ^25^Mg^2+^ uptake was inhibited. When subjected to 24 hours ^25^Mg^2+^ loading, *Cnnm2*-expressing cells showed a significantly higher ^25^Mg^2+^ content baseline. Subsequently, 15 minutes extrusion of *Cnnm2*-expressing cells demonstrated no difference in Mg^2+^ extrusion rate, compared to mock-transfected cells (Figure 2G).

### Mutations impair CNNM2-dependent Mg^2+^ uptake

**Figure 3:**
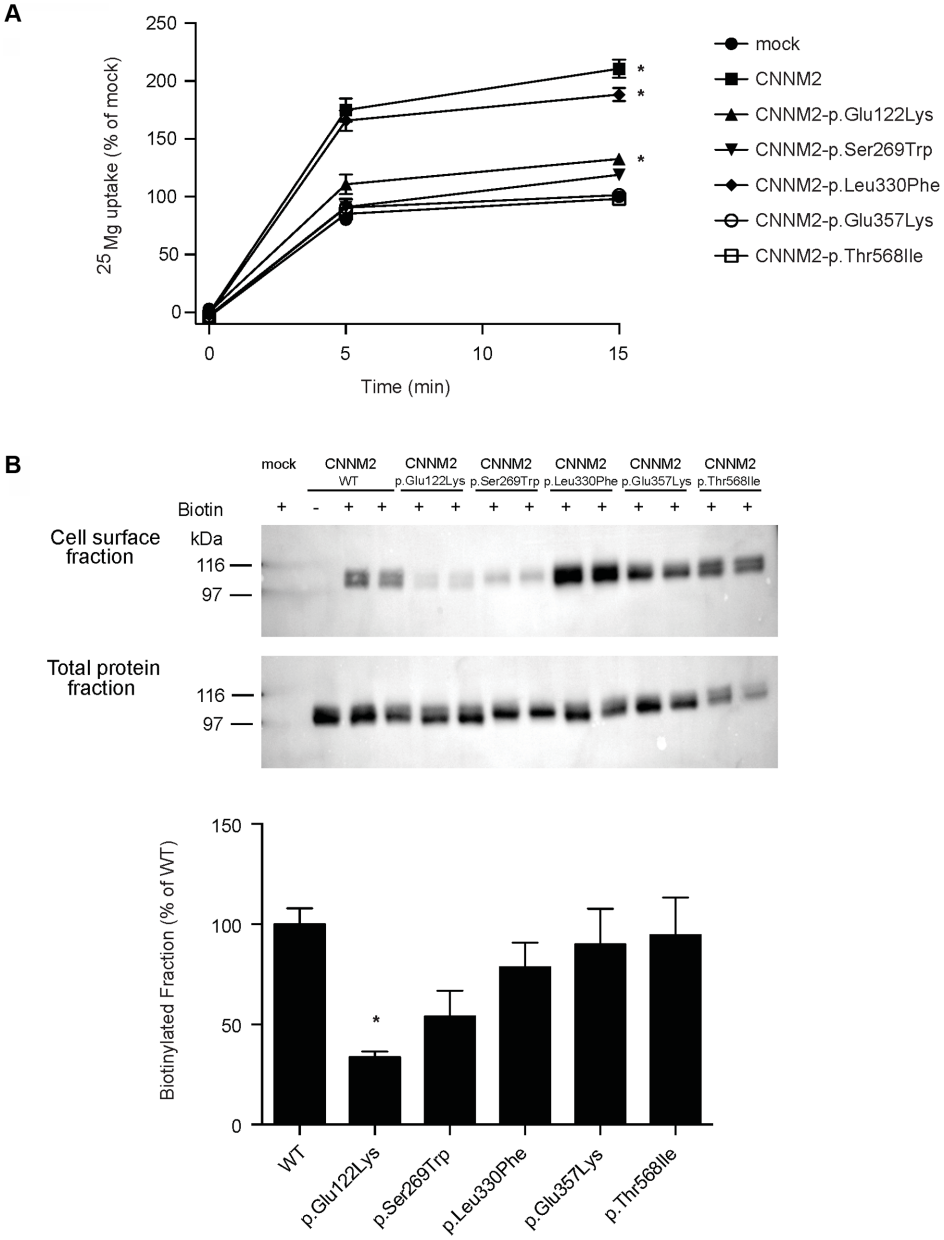
CNNM2 mutations impair Mg^2+^ uptake in HEK293 cells. (A) Time curve of ^25^Mg^2+^ uptake in mock, wild-type CNNM2 and mutant CNNM2 transfected cells. Symbols indicate cells transfected with the vector empty (• mock) or containing *Cnnm2* sequences encoding for wild-type or mutant CNNM2 proteins (▪ CNNM2, ▴ CNNM2-p.Glu122Lys, ▾ CNNM2-p.Ser269Trp, ⧫ CNNM2-p.Leu330Phe, ○ CNNM2-p.Glu357Lys, □ CNNM2-p.Thr568Ile). Each data point represent the mean of 3 independent experiments ± SEM. * indicates significant differences compared to mock (*P*<0.05). (B) Representative immunoblots showing that p.Glu122Lys and p.Ser269Trp mutations reduce CNNM2 membrane expression (upper blot) and a CNNM2 expression control (lower blot). Quantification of cell surface expression of wild-type (WT) and mutant CNNM2 proteins corrected for total protein expression. Results are the mean ± SEM of 3 independent experiments. * indicate significant differences compared to WT CNNM2 transfected cells (*P*<0.05).

To characterize the effect of the *CNNM2* mutations identified in our hypomagnesemic patients, ^25^Mg^2+^ uptake was determined in HEK293 cells expressing mutant CNNM2 proteins. Of all missense mutations that are identified to date, only p.Leu330Phe was capable of increasing ^25^Mg^2+^ uptake to a similar extent as wild-type CNNM2 (Figure 3A). All other CNNM2 mutants exhibited severely decreased ^25^Mg^2+^ uptake or had lost their ability to increase ^25^Mg^2+^ uptake completely. To examine whether CNNM2 dysfunction can be explained by a reduced plasma membrane expression, all mutants were subjected to cell surface biotinylation analysis. Indeed, p.Glu122Lys CNNM2 membrane expression was significantly reduced compared with wild-type CNNM2 (66% decrease, *P*<0.05) and p.Ser269Trp CNNM2 showed a trend towards reduction (46% decrease, Figure 3B).

### Disturbed Mg^2+^ homeostasis and brain abnormalities in *cnnm2* morphant zebrafish larvae

Patients with mutations in *CNNM2* suffer from hypomagnesemia. Therefore, zebrafish *cnnm2* morphants were tested for disruptions of their Mg^2+^ homeostasis. Extraction of serum from zebrafish embryos is technically not feasible. Thus, total body Mg contents of controls and morphant larvae were examined at 5 days post-fertilization (dpf). During these 5 days of zebrafish development, intestinal absorption of Mg^2+^ does not take place since larvae do not eat and drink. For that reason, Mg^2+^ homeostasis is the result of the balance between Mg^2+^ excretion, passive Mg^2+^ uptake from the yolk, Mg^2+^ reabsorption in the kidney, and Mg^2+^ uptake in the integument, where ionocytes are analogous to renal tubular cells in terms of function and transporter and channel expression [16]. Therefore, when knocking down a gene involved in active epithelial Mg^2+^ uptake, disturbances in total body Mg content reliably represent disturbances in active Mg^2+^ reabsorption and/or uptake, through pronephric (renal) tubular cells and/or their analogous in the skin, respectively.

**Figure 4:**
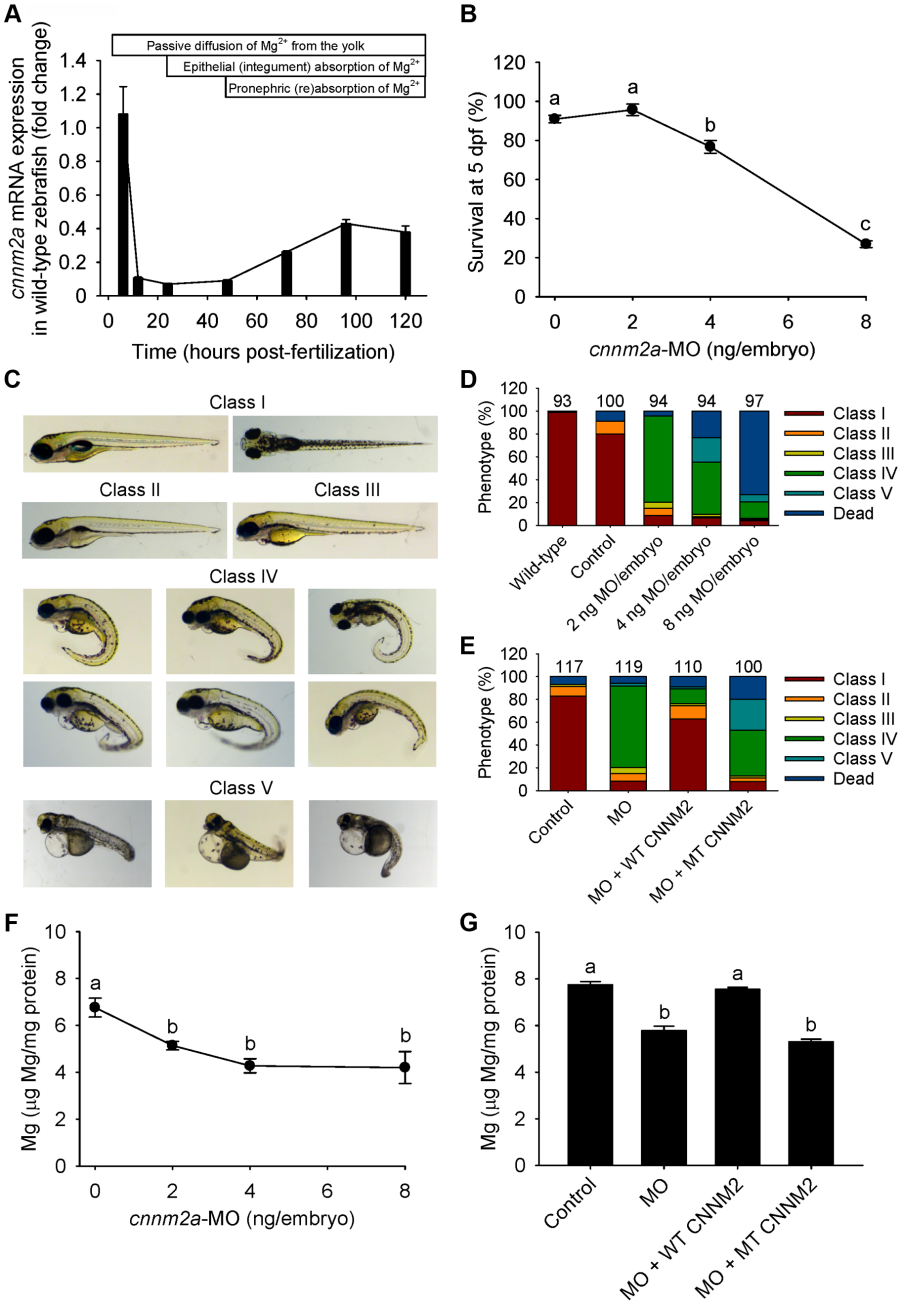
Knockdown of *cnnm2a* results in Mg wasting in zebrafish larvae (5 dpf). (A) mRNA expression of *cnnm2a* in developing zebrafish. Expression patterns were analysed by RT-qPCR (n = 6 per time point). (B) Survival curve at 5 dpf (n = 3 per experimental condition). The dose of zero represents injection with control-MO. (C) Morphological phenotypes in zebrafish larvae (5 dpf) in *cnnm2a* knockdown experiments. (D) Distribution of morphological phenotypes in zebrafish larvae (5 dpf) untreated (wild-type) or injected with different doses of *cnnm2a*-MO or control-MO. Numbers on top of the bars indicate the number of animals in each experimental condition. (E) Distribution of morphological phenotypes in zebrafish larvae at 5 dpf in rescue experiments. The wild-type phenotype (class I) was restored in morphants by co-injection of *cnnm2a*-MO (2 ng MO/embryo) with wild-type (WT) CNNM2 cRNA (50 pg cRNA/embryo), but not with mutant (MT, p.Glu357Lys) CNNM2 cRNA (50 pg cRNA/embryo). (F) Magnesium content in zebrafish injected with different doses of *cnnm2a*-MO, the dose of zero represents injection with control-MO (n = 10 per experimental condition except in 8 ng MO-injected zebrafish where n = 5). (G) Rescue of Mg wasting in morphant zebrafish by co-injection of *cnnm2a*-MO (2 ng MO/embryo) with cRNA encoding for wild-type (WT) CNNM2 (50 pg cRNA/embryo). Co-injection with cRNA encoding for mutant (MT, p.Glu357Lys) CNNM2 (50 pg cRNA/embryo) did not restore Mg levels (n = 10 per experimental condition). Data are presented as mean ± SEM. Different letters indicate significant differences between mean values in experimental groups (*P*<0.05).

The *cnnm2a* gene, one of the two zebrafish *cnnm2* paralogues, is expressed during early development (Figure 4A). Injection in embryos of higher doses than 2 ng of morpholino (MO) blocking *cnnm2a* translation resulted in a significantly reduced survival compared to controls at 5 dpf (Figure 4B). At non-lethal doses of MO (when mortality caused by the *cnnm2a*-MO does not differ significantly from mortality in controls), knockdown of *cnnm2a* resulted in morphological phenotypes characterized by enlarged pericardial cavities and notochord defects (Figure 4C–D). The biochemical equivalency between mammalian CNNM2 and its zebrafish orthologue *cnnm2a* was demonstrated by the fact that co-injection of *cnnm2a*-MO with mouse wild-type *Cnnm2* cRNA induced a rescue of all phenotypes observed (Fig. 4E). Conversely, co-injection with mouse mutant *Cnnm2* cRNA did not result in any rescue. In line with the symptom of hypomagnesemia in patients with mutated *CNNM2*, *cnnm2a* morphants exhibited significantly reduced levels of Mg compared to controls when increasing doses of MO were injected (Figure 4F). Total Mg content in *cnnm2a* morphants was restored to control levels when *cnnm2a*-MO was co-injected with cRNA encoding for mouse wild-type CNNM2 and not with mutant *Cnnm2* (Figure 4G). This demonstrated that the defects observed in morphants were indeed caused by dysfunctional *cnnm2a* and not by toxic off-target effects.

**Figure 5:**
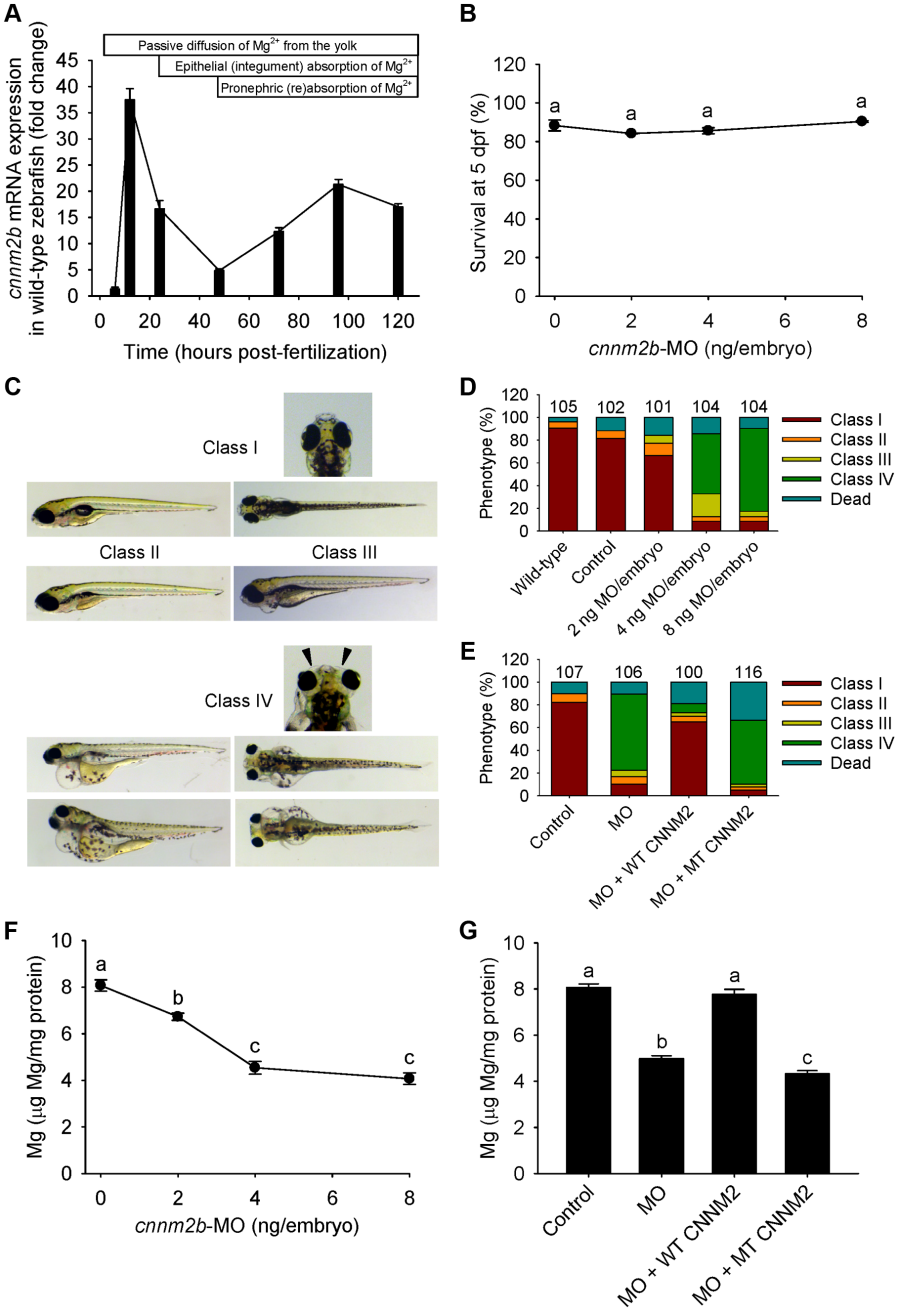
Knockdown of *cnnm2b* results in Mg wasting and brain malformations in zebrafish larvae (5 dpf). (A) mRNA expression of *cnnm2b* in developing zebrafish. Expression patterns were analysed by RT-qPCR (n = 6 per time point). (B) Survival curve at 5 dpf (n = 3 per experimental condition). The dose of zero represents injection with control-MO. (C) Morphological phenotypes in zebrafish larvae (5 dpf) in *cnnm2b* knockdown experiments. (D) Distribution of morphological phenotypes in zebrafish larvae (5 dpf) untreated (wild-type) or injected with different doses of *cnnm2b*-MO or control-MO. Brain malformations (widened cerebrospinal fluid spaces, class IV phenotype) are prominent in morphants injected with 4–8 ng MO/embryo. Numbers on top of the bars indicate the number of animals in each experimental condition. (E) Distribution of morphological phenotypes in zebrafish larvae at 5 dpf in rescue experiments. The wild-type phenotype (class I) was restored in morphants by co-injection of *cnnm2b*-MO (8 ng MO/embryo) with wild-type (WT) CNNM2 cRNA (50 pg cRNA/embryo), but not with mutant (MT, p.Glu357Lys) CNNM2 cRNA (50 pg cRNA/embryo). (F) Magnesium content in zebrafish injected with different doses of *cnnm2b*-MO. The dose of zero represents injection with control-MO (n = 10 per experimental condition). (G) Rescue of Mg wasting in morphant zebrafish by co-injection of *cnnm2b*-MO (8 ng MO/embryo) with cRNA encoding for wild-type (WT) CNNM2 (50 pg cRNA/embryo). Co-injection with cRNA encoding for mutant (MT, p.Glu357Lys) CNNM2 (50 pg cRNA/embryo) did not restore Mg levels (n = 10 per experimental condition). Data are presented as mean ± SEM. Different letters indicate significant differences between mean values in experimental groups (*P*<0.05).

Zebrafish *cnnm2b* is also expressed during early development (Figure 5A). Survival in *cnnm2b* morphants was not affected by the knockdown (Figure 5B). The *cnnm2b* morphants were characterized by enlarged pericardial cavities, kidney cysts and, in agreement with the morphological brain abnormalities observed in the F1.1 patient, by accumulation of cerebrospinal fluid in the cerebrum (Figure 5C–D). Interestingly, most *cnnm2b* morphants were morphologically normal at the dose of 2 ng MO/embryo (Figure 5D). All morphological phenotypes were rescued by co-injection of *cnnm2b*-MO with mouse wild-type *Cnnm2* (Figure 5E). As for *cnnm2a*, *cnnm2b* was demonstrated to reduce Mg levels when knocked down (Figure 5F) and to be functionally equivalent to mammalian *CNNM2* in cRNA rescue experiments (Figure 5G). Phenotype rescue with mouse wild-type *Cnnm2* demonstrated the absence of toxic off-target effects and the specificity of the *cnnm2b*-MO to produce defects attributable to impaired *CNNM2* function.

### Brain abnormalities and increased spontaneous contractions in *cnnm2* morphant zebrafish embryos

**Figure 6:**
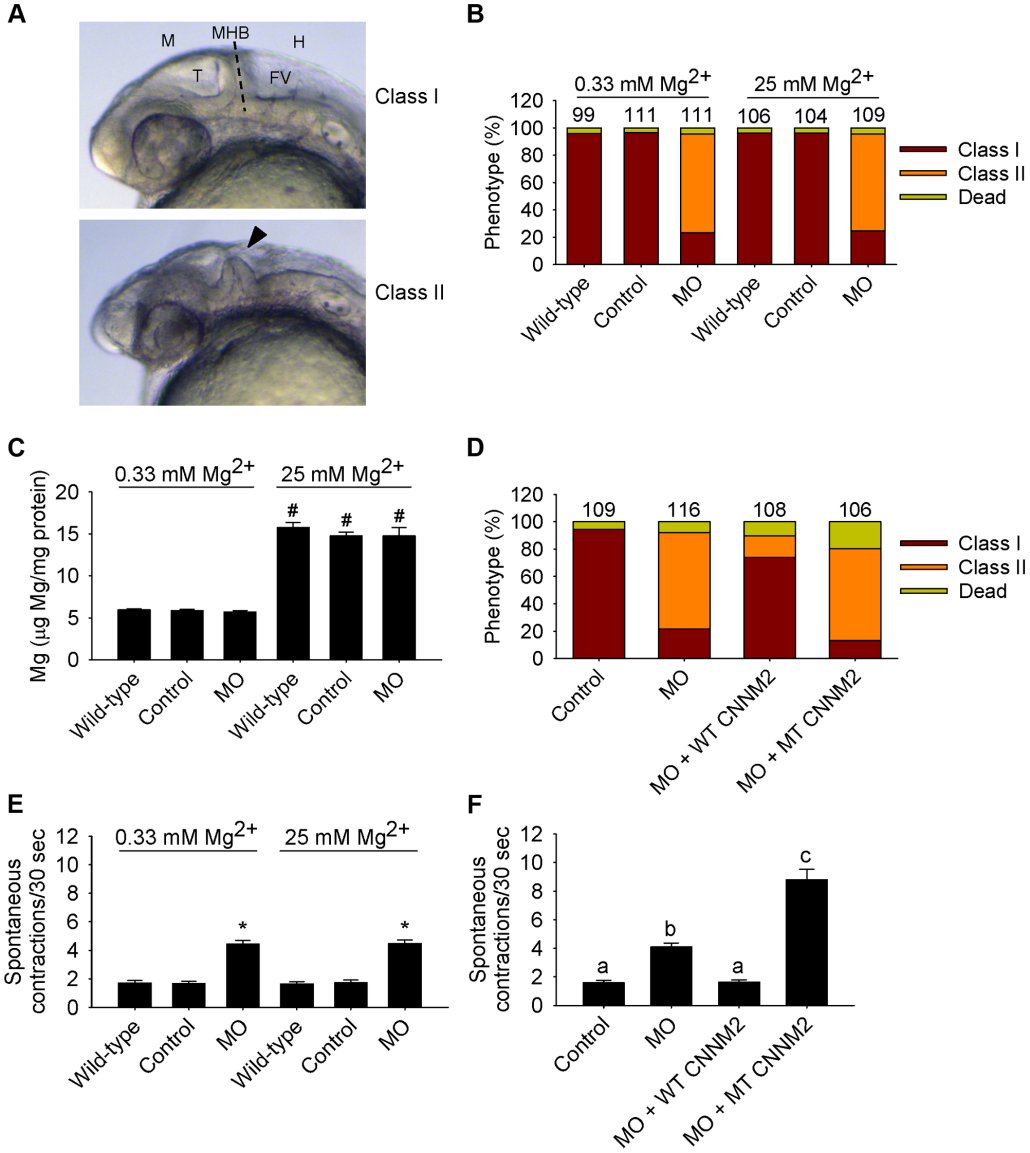
Dysfunctional c*nnm2a* causes brain abnormalities and increased spontaneous contractions in zebrafish embryos (25 hpf). (A) Phenotypes in zebrafish embryos untreated (wild-type) or following treatment with *cnnm2a*-MO (2 ng MO/embryo) or control-MO. Abbreviations indicate the following parts in the zebrafish embryonic brain: M, midbrain; T, tectum; MHB, midbrain-hindbrain boundary; FV, fourth ventricle; and H, hindbrain. (B) Distribution of phenotypes and (C) Mg content (n = 10 per experimental condition) in zebrafish embryos untreated (wild-type) or injected with 2 ng of *cnnm2a*-MO or control-MO and exposed to a medium with a concentration of Mg^2+^ of 0.33 or 25 mM. Numbers on top of the bars indicate the number of animals in each experimental condition. (D) Restoration of normal brain development by co-injection of *cnnm2a*-MO (2 ng MO/embryo) with cRNA encoding for wild-type (WT) CNNM2 (50 pg cRNA/embryo), and not by co-injection with cRNA encoding for mutant (MT, p.Glu357Lys) CNNM2 (50 pg cRNA/embryo). (E) Spontaneous contractions in zebrafish embryos untreated (wild-type) or injected with 2 ng of *cnnm2a*-MO or control-MO and exposed to a medium with a concentration of Mg^2+^ of 0.33 or 25 mM (n = 30 per experimental condition). (F) Restoration of normal spontaneous contraction activity (n = 30 per experimental condition) by co-injection of *cnnm2a*-MO (2 ng MO/embryo) with cRNA encoding for wild-type (WT) CNNM2 (50 pg cRNA/embryo), and not by co-injection with cRNA encoding for mutant (MT, p.Glu357Lys) CNNM2 (50 pg cRNA/embryo). Data are presented as mean ± SEM. *P<0.05 *versus* wild-type and control. ^#^ *P*<0.05 *versus* Mg^2+^-normal (0.33 mM Mg^2+^) medium. Data are presented as mean ± SEM. Different letters indicate significant differences between mean values in experimental groups (*P*<0.05).

Patients with *CNNM2* mutations suffer from mental retardation and seizures. As the severe neurological phenotype in patients F1.1 and F1.2 was diagnosed early after birth (Table 1), we hypothesized that the deleterious effects of mutant *CNNM2* could result from early developmental defects in brain primordia. In zebrafish, the segmental organization of the brain rudiment, and morphologically visible boundaries and primordia are established at 25 hours post-fertilization (hpf). At this stage, maldevelopment of the midbrain hindbrain boundary (MHB) is observed in *cnnm2a* morphant embryos (Figure 6A–B). Interestingly, these phenotypes could not be rescued by exposure to media with high Mg^2+^ concentrations (Figure 6B), even though these media significantly increased the Mg content of morphant embryos (Figure 6C). More importantly, phenotypes were rescued by co-injection with the mouse orthologue cRNA and not by co-injection with the mutant transcript (Figure 6D). In addition to brain developmental defects, the frequency of spontaneous embryonic contractions was increased in *cnnm2a* morphants compared to controls (Figure 6E), which could indicate that (motor) neurons are hyperexcitable [26]. This phenotype was not rescued by exposure to high Mg^2+^ concentrations in the medium (Figure 6E). In contrast, co-injection of the *cnnm2a*-MO with mouse wild-type *Cnnm2* cRNA did result in a rescue of the neurological functioning (Figure 6F). Conspicuously, co-injection with the mutant *Cnnm2* cRNA even worsened this motor neuronal phenotype by increasing the number of spontaneous contractions significantly compared to embryos injected only with *cnnm2a*-MO (Figure 6F; Movies S1, S2, S3).

**Figure 7:**
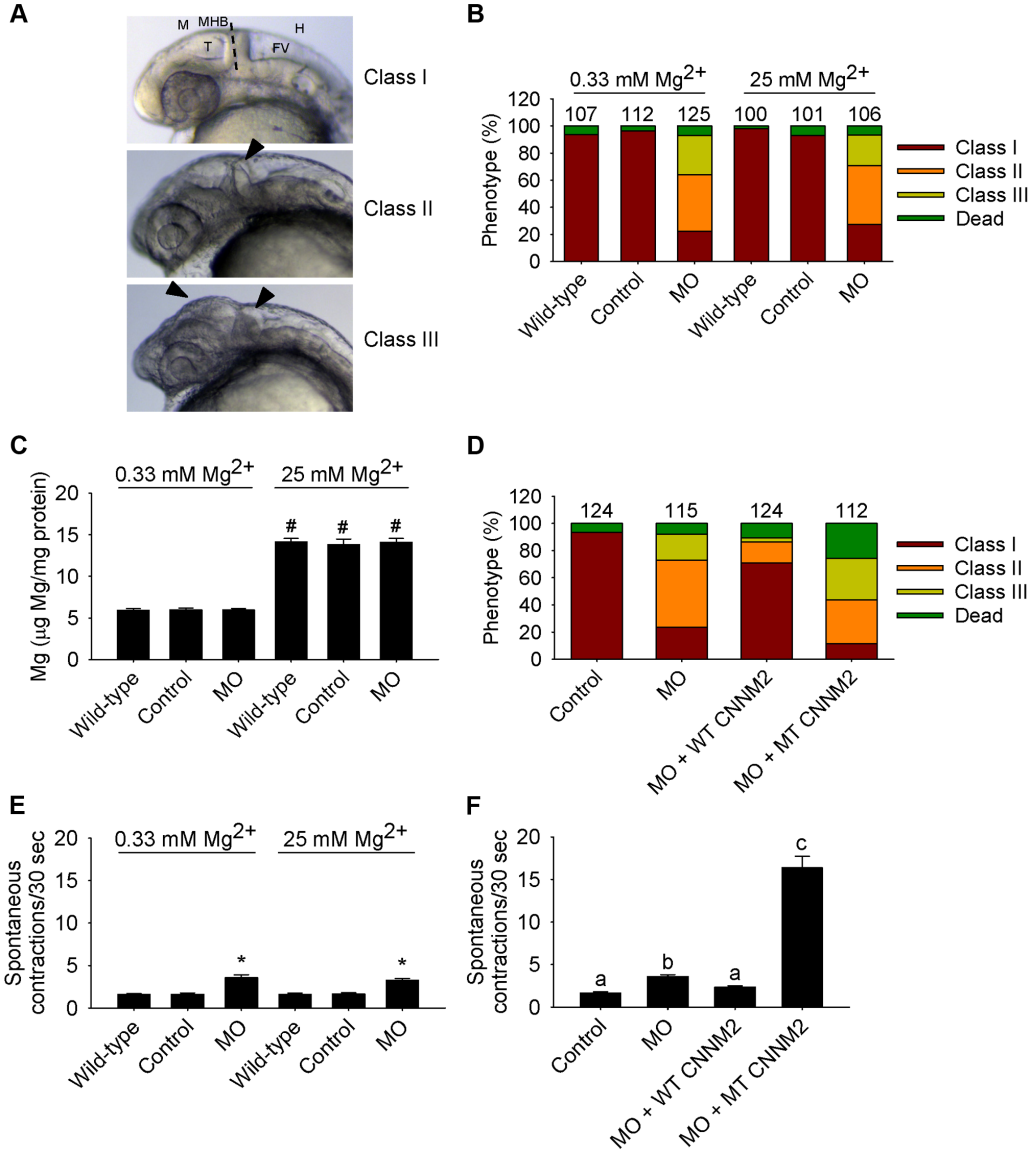
Dysfunctional c*nnm2b* causes brain abnormalities and increased spontaneous contractions in zebrafish embryos (25 hpf). (A) Phenotypes in zebrafish embryos untreated (wild-type) or following treatment with *cnnm2b*-MO (8 ng MO/embryo) or control-MO. See Figure 6 for an explanation of the abbreviations shown. (B) Distribution of phenotypes and (C) Mg content (n = 10 per experimental condition) in zebrafish embryos untreated (wild-type) or injected with 8 ng of *cnnm2b*-MO or control-MO and exposed to a medium with a concentration of Mg^2+^ of 0.33 or 25 mM. Numbers on top of the bars indicate the number of animals in each experimental condition. (D) Restoration of normal brain development by co-injection of *cnnm2b*-MO (8 ng MO/embryo) with cRNA encoding for wild-type (WT) CNNM2 (50 pg cRNA/embryo), and not by co-injection with cRNA encoding for mutant (MT, p.Glu357Lys) CNNM2 (50 pg cRNA/embryo). (E) Spontaneous contractions in zebrafish embryos untreated (wild-type) or injected with 8 ng of *cnnm2b*-MO or control-MO and exposed to a medium with a concentration of Mg^2+^ of 0.33 or 25 mM (n = 30 per experimental condition). (F) Restoration of normal spontaneous contraction activity (n = 30 per experimental condition) by co-injection of *cnnm2b*-MO (8 ng MO/embryo) with cRNA encoding for wild-type (WT) CNNM2 (50 pg cRNA/embryo), and not by co-injection with cRNA encoding for mutant (MT, p.Glu357Lys) CNNM2 (50 pg cRNA/embryo). Data are presented as mean ± SEM. *P<0.05 *versus* wild-type and control. ^#^ *P*<0.05 *versus* Mg^2+^-normal (0.33 Mm Mg^2+^) medium. Data are presented as mean ± SEM. Different letters indicate significant differences between mean values in experimental groups (*P*<0.05).

In the case of *cnnm2b* morphants, enlarged tectums were also present in a 30% of morphants in addition to the defects in the MHB, phenotypes that were not rescued by exposure to high Mg^2+^ concentrations (Figure 7A–C) but by co-injection of *cnnm2b*-MO with mouse wild-type *Cnnm2* cRNA (Figure 7D). Spontaneous contraction frequency was increased in *cnnm2b* morphants (Figure 7E), restored to control levels with overexpression of mouse wild-type *Cnnm2* (Figure 7F), and 4-fold increased with overexpression of mouse mutant *Cnnm2* compared to *cnnm2b* morphants injected solely with *cnnm2b*-MO (Figure 7F; Movies S4, S5, S6).

The validated cRNA rescue controls proved the specificity of the brain defects observed, attributable to dysfunctional orthologues of *CNNM2* for both translation blocking MOs used.

### Weaker touch-evoked escape behaviour in *cnnm2* morphant zebrafish larvae

As our *in vitro* data pointed to a putative interaction between CNNM2 and TRPM7 and zebrafish morphants presented brain developmental defects, the touch-evoked escape behaviour in zebrafish was evaluated, a parameter largely dependent on TRPM7 activity in sensory neurons and/or brain development [27]–[29]. Indeed, in *cnnm2a* and *cnnm2b* morphants (at 5 dpf), touch-evoked escape behaviour was significantly weaker than that in controls (Figures S1, S2). Additionally, this phenotype was rescued by co-injection of the MO with wild-type *Cnnm2* cRNA and not by mutant *Cnnm2* cRNA. As for the other phenotypes, cRNA rescues proved the causality between the weak touch-evoked escape behaviour in morphants and dysfunctional *cnnm2* paralogues.

## Discussion

In the present study, a severe brain phenotype consisting of cerebral seizures, mental retardation and brain malformations in patients with hypomagnesemia was shown to be caused by mutations in *CNNM2*. Our experiments established *CNNM2* as a new essential gene in brain development, neurological functioning and Mg^2+^ homeostasis. This notion is supported by the following observations; *i*) hypomagnesemic patients with *CNNM2* mutations suffer from seizures, mental disability, and if mutations are present in recessive state, brain malformations are observed in addition; *ii*) Mg^2+^ supplementation does not improve the neurological phenotype of the patients; *iii*) CNNM2 increases Mg^2+^ uptake in HEK293 cells, whereas mutant CNNM2 does not; *iv*) knockdown of CNNM2 orthologues in zebrafish results in impaired development of the brain, abnormal neurodevelopmental phenotypes manifested as altered locomotor and touch-evoke escape behaviours, and Mg wasting; *v*) the zebrafish phenotype can be rescued by injection of mouse *Cnnm2* cRNA.

In addition to the previously reported dominant mode of inheritance [9], the genetic findings in our patients support heterogenous patterns of inheritance. In family F1, a recessive mode of *CNNM2* inheritance was observed. The homozygous CNNM2 p.Glu122Lys mutation in this family resulted in the manifestation of a neonatal onset and a considerably more severe cerebral involvement than in the remaining patients. Yet, a central nervous system (CNS) phenotype with seizures and intellectual disability, which was not reported previously, represented the cardinal clinical symptom in all of our patients. Seizures constituted the major symptom at manifestation coinciding with hypomagnesemia, but were also seen during follow-up despite Mg^2+^ supplementation. Pronounced Mg^2+^ deficiency reflected by severely low serum Mg^2+^ levels clearly represents a promotive element in the development of seizures. However, the persistence of seizure activity despite Mg^2+^ supplementation might point to a genuine disturbance in brain function caused by defective CNNM2. Accordingly, the extent of hypomagnesemia found in the two siblings with the recessive mutation, F1.1 and F1.2, was identical to other patients (F2.1–F5.1) with heterozygous CNNM2 mutations and a milder neurological phenotype. Continuous oral Mg^2+^ supplementation stabilized serum Mg^2+^ levels in the subnormal range, however a complete normalization of Mg^2+^ metabolism could not be achieved in any of the patients.

In three out of five families (F2, F3 and F4), the mutation of the patient was not present in the parents. This finding supports the recent advancements evidencing that *de novo* mutations provide an important mechanism in the development of mental disability disorders [30]. Remarkably, the same *de novo* p.Glu357Lys mutation was identified in two unrelated individuals. Although the mutation rate along the human genome varies significantly [31], the chance to observe an identical *de novo* base pair change in two individuals is extremely small, supporting causality of this mutation. The clinical and genetic findings observed in patient F5.1 should be interpreted with caution. Though the p.Leu330Phe variant was not present in controls or exome variant databases, the presence of an innocuous polymorphism cannot be completely excluded. The clinical phenotype with a milder degree of intellectual disability and a later manifestation with hypomagnesemic symptoms during adolescence support a partial loss of CNNM2 function caused by the p.Leu330Phe variant. Unfortunately, the patient was not available for clinical re-evaluation.

Over the recent years, the function of CNNM2 in the context of Mg^2+^ handling has been heavily debated [9], [11]–[13], [20]. Therefore, an important question is how CNNM2 mutations cause impaired Mg^2+^ metabolism and lead to CNS dysfunction. Functional studies in HEK293 cells demonstrated a putative role in cellular Mg^2+^ transport. Overexpression of CNNM2 increased cellular Mg^2+^ uptake, which was abrogated by introduction of the CNNM2 mutants identified in our patients. The p.Ser269Trp and p.Glu357Lys mutants as well as the previously published p.Thr568Ile mutant [9], all identified in heterozygous state, failed to enhance the cellular Mg^2+^ uptake, indicating a loss-of-function in mutated CNNM2. The recessively inherited p.Glu122Lys mutant identified in patients F1.1 and F1.2 displayed a small but significant residual function, while Mg^2+^ uptake was almost completely retained for mutant p.Leu330Phe. Biotinylation experiments demonstrated a trafficking defect of p.Glu122Lys and p.Ser269Trp mutants supporting a loss-of-function nature of CNNM2 mutations. Together, these findings argue for distinct degrees of severity of the disease depending on the number of affected alleles. Furthermore, a small residual function of p.Glu122Lys is in line with the lack of a clinical phenotype in the parents of family F1. The parents, however, declined a thorough evaluation of their Mg^2+^ status.

To further analyze the relevance of CNNM2 for brain and Mg^2+^ metabolism deduced from the human disease model, the translation of orthologues of *CNNM2* (*cnnm2a* and *cnnm2b*) was knocked down in zebrafish. In line with the human disease, the concentration of total body Mg was decreased in zebrafish *cnnm2a* and *cnnm2b* morphants when compared to controls. The decrease in total body Mg content is interpreted as a decrease in the renal absorption and/or skin uptake (through ionocytes analogous to renal tubular cells) of the ionic fraction, Mg^2+^, since only Mg^2+^ is transported transcellularly and no intestinal Mg^2+^ uptake takes place in zebrafish larvae. Additionally, Mg losses observed in morphant larvae were rescued by expression of wild-type *Cnnm2*, but not by expression of mutant *Cnnm2*. This demonstrates the specificity of our MO antisense oligos, as well as the functional equivalence between mammalian CNNM2 and its zebrafish orthologues.

Consistent with the human pathology, knockdown of *cnnm2a* or *cnnm2b* induced brain malformations. Specifically, the brain phenotype observed in *cnnm2b* morphants resembles that found in patient F1.1 showing enlarged outer cerebrospinal liquor spaces. This provides further consistency to link CNNM2 dysfunction with the brain morphological defects found in this homozygous patient. The absence of outer cerebrospinal liquor spaces in the cerebrum of *cnnm2a* morphants shows that *cnnm2* paralogues in zebrafish are a case of subfunctionalization at the level of the cerebrum. CNS malformations were rescued in morphants by co-injection with mouse wild-type *Cnnm2*.

In homozygous patients, the neurological defects became evident early after birth. In line with a developmental role for CNNM2 within the CNS, gene expression of zebrafish *cnnm2a* and *cnnm2b* peaked within the first 24 hpf. In addition, *in situ* hybridization located *cnnm2a* expression specifically in the MHB [32], an organizing center in the neural tube that determines neural fate and differentiation in the CNS during development [33]–[34]. Indeed, the most striking brain developmental defect in our study is maldevelopment of the MHB. These defects were rescued with *Cnnm2* cRNA. Interestingly, the brain phenotypes observed in both zebrafish and patients were independent of Mg^2+^, as Mg^2+^ supplementation was unsuccessful to rescue the phenotypes. Thus, our findings suggest that a brain-specific CNNM2 function is crucial for the development of constitutive regions of the CNS, which in the zebrafish model is illustrated by defects in the MHB.

At 25 hpf, a time point in which the locomotor behaviour is unaffected by the brain and only depends on signals from the spinal cord [27], zebrafish morphant embryos displayed an increased frequency of spontaneous contractions, especially when the MOs were co-injected with mutant *Cnnm2*. This hyperexcitability of motor neurons suggests a function of zebrafish Cnnm2 proteins in the regulation of the activity of the neurological network in the spinal cord or in the synaptic junctions with muscle fibbers. Consistent with these findings, patients with mutations in *CNNM2* presented impaired motor skills, which were severe in the case of homozygous patients.

In the CNS, TRPM7 is essential during early development [35], as it modulates neurotransmitter release in sensory neurons [36]–[37]. Specifically, when using 2-APB, an inhibitor of TRPM7 [22], CNNM2-dependent Mg^2+^ transport was abolished in HEK293 cells. Remarkably, in a similar fashion to *trpm7* mutants in zebrafish [28]–[29], *cnnm2a* or *cnnm2b* morphants showed weaker touch-evoked escape behaviour compared to controls. In 5 dpf larvae, and unlike in 25 hpf embryos, locomotor behaviours elicited by touch require the involvement of high brain structures [27]. Therefore, it is reasoned that CNNM2 conditions locomotor behaviour with an etiology that can be related to lack of excitation of sensory neurons *via* TRPM7 and/or to the defects in early brain development observed in zebrafish morphant embryos. In kidney, where CNNM2 is expressed at the basolateral membrane in DCT, specific regulation of TRPM7 Mg^2+^ reabsorption is unlikely, since TRPM6 is the main Mg^2+^ transporter in this segment. TRPM7 is a ubiquitously expressed gene regulating cellular Mg^2+^ metabolism, which is for instance involved in regulation of brain Mg^2+^ levels [6]. Therefore, one could hypothesize that CNNM2 may regulate other proteins in addition to TRPM7 in kidney for the control of Mg^2+^ reabsorption, which remain to be identified.

In conclusion, our findings of *CNNM2* mutations in patients with hypomagnesemia and severe neurological impairment widen the clinical spectrum of CNNM2-related disease. By establishing a zebrafish CNNM2 loss-of-function model of the genetic disease, we provide a unique model for the testing of novel therapeutic drugs targeting CNNM2.

## Materials and Methods

### Ethics statement

All genetic studies were approved by the ethics committee of the Westfälische Wilhelms University, Münster. All patients or their parents provided written informed consent in accordance to the Declaration of Helsinki. All animal experiments were performed in agreement with European, National and Institutional regulations. Animal experimentation and analysis was restricted to the first five days post-fertilization (dpf).

### Patients

We studied a cohort of six patients from five families with hypomagnesemia and mental retardation. Patients F1.1 to F4.1 are followed in secondary or tertiary care neuropediatric centres. Neuroimaging was performed in F1.1, F2.1, F3.1, and F4.1 by cranial MRI (magnetic resonance imaging). Psychological diagnostic evaluation in patients F2.1 and F3.1 was performed using Snijders Oomen Non-Verbal (SON) Intelligence Test (revised) 5.5–17 years. Copy number variations (CNVs) associated with neurodevelopmental delay and intellectual disability were excluded in patients F1.1 and F2.1 by array CGH (comparative genomic hybridization) using the Sureprint G3 Human CGH Microarray kit (Agilent Technologies, Boeblingen, Germany) in patient F1.1 and using the Affymetrix Cytogenetics Whole-Genome 2.7 Array in patient F2.1.

### Homozygosity mapping and CNNM2 mutational analysis

Genomic DNA of affected individuals and available family members was extracted from whole blood using standard methods. A genome scan for shared homozygous regions was performed in the two affected children F1.1 and F1.2 with suspected parental consanguinity. Samples were genotyped on an Illumina human 660W Quad beadchip SNP array (Illumina, Eindhoven, The Netherlands). Merlin 1.1.2 (University of Michigan, Ann Arbor, MI, USA) was used to determine homozygous regions by linkage analysis. As exact information on pedigree structure was missing, we used a 1.7 Mb threshold for regions identical by descent that is very rarely crossed by non-consanguineous samples, but allows to identify most of the true homozygosity regions if parental consanguinity is present [38]. A list of candidate genes within the identified homozygous intervals was generated including known Refseq genes as well as novel transcripts using Ensembl Genome assembly GRCh37 via biomart (www.ensembl.org). At a cut-off size of >1.7 Mb, eleven critical intervals were yielded on autosomes with a cumulative size of 62 Mb. The gene list generated from these loci included 322 RefSeq genes and putative transcripts, including *CNNM2* in a critical interval of 7.1 Mb on chromosome 10. The entire coding region and splice-sites of the most promising candidate gene *CNNM2* were sequenced from both strands (Genbank: NM_017649.4, Uniprot: Q9H8M5). After discovery of a homozygous mutation in the index family F1, the mutational screening was extended to patients with hypomagnesemia without mutations in known genes involved in hereditary magnesium wasting. The presence of newly identified *CNNM2* sequence variations was tested in at least 204 ethnically matched control alleles and compared to publically available exome data (www.1000genomes.org; evs.gs.washington.edu). Additionally, all identified mutations were ranked for potential damage on protein function using Polyphen-2 (genetics.bwh.harvard.edu/cgi-bin/ggi/ggi2.cgi).

### DNA constructs

Mouse wild-type *Cnnm2* construct was cloned into the pCINeo HA IRES GFP vector as described previously [12]. *Cnnm2* mutations were inserted in the construct using the QuikChange site-directed mutagenesis kit (Stratagene, La Jolla, CA, USA) according to the manufacturer's protocol. All constructs were verified by sequence analysis. Primer sequences used for cloning or mutagenesis PCR are reported in Table S1.

### Cell culture

HEK293 cells were grown in Dulbecco's modified eagle's medium (DMEM, Bio Whittaker-Europe, Verviers, Belgium) containing 10% (v/v) fetal calf serum (PAA, Liz, Austria), 2 mM L-glutamine and 10 µg/mL non-essential amino acids, at 37°C in a humidity-controlled incubator with 5% (v/v) CO_2_. The cells were transiently transfected with the respective DNA constructs using Lipofectamin 2000 (Invitrogen, Breda, The Netherlands) at 1∶2 DNA∶Lipofectamin ratio for 48 hours unless otherwise stated.

### Cell surface biotinylation

HEK293 cells were transfected with wild-type and mutant CNNM2 constructs for 48 hours. Subsequently, cell surface proteins were biotinylated as described previously [39]. Briefly, cell surface proteins were biotinylated for 30 min at 4°C in 0.5 mg/mL sulfo-NHS-LC-LC-biotin (Pierce, Rockford, IL, USA). Cells were washed and lysed in lysis buffer (150 mM NaCl, 5 mM EGTA, Triton 1% (v/v), 1 µg/ml pepstatin, 1 mM PMSF, 5 µg/ml leupeptin, 5 µg/ml aproptin, 50 mM Tris/HCl, pH 7.5). 10% (v/v) of the sample was taken as input control and the rest of the protein lysates were incubated overnight with NeutrAvidin-agarose beads (Pierce, Rockford, IL, USA) at 4°C. The next day, unbound protein was discarded by washing the beads 5 times with lysis buffer. The remaining protein lysates were denatured in Laemmli containing 100 mM DTT for 30 min at 37°C and subsequently subjected to SDS-PAGE. Then, immunoblots were incubated with mouse anti-HA 1∶5,000 primary antibodies (Cell Signaling Technology, Danvers, MA, USA) and peroxidase conjugated sheep anti-mouse secondary antibodies 1∶10,000 (Jackson Immunoresearch, Suffolk, UK).

### Magnesium transport assays

HEK293 cells were transfected with wild-type and mutant CNNM2 constructs for 48 hours and seeded on poly-L-lysine (Sigma, St Louis, MO, USA) coated 12-well plates. Mg^2+^ uptake was determined using a stable ^25^Mg^2+^ isotope (Cortecnet, Voisins Le Bretonneux, France), which has a natural abundance of ±10%. Cells were washed with basic uptake buffer (125 mM NaCl, 5 mM KCl, 0.5 mM CaCl_2_, 0.5 mM Na_2_HPO_4_, 0.5 mM Na_2_SO_4_, 15 mM HEPES/NaOH, pH 7.5) and subsequently placed in basic uptake buffer containing 1 mM ^25^Mg^2+^ (purity ±98%) for 5 minutes unless stated differently. After washing three times with ice-cold PBS, the cells were lysed in HNO_3_ (≥65%, Sigma) and subjected to ICP-MS (inductively coupled plasma mass spectrometry) analysis. For extrusion experiments, cells were transfected with wild-type or mutant CNNM2 constructs for 24 hours. After 24 hours, cells were placed in culture medium containing 1 mM ^25^Mg^2+^ (purity ±98%) for an additional 48 hours. Before the start of the experiment, the cells were briefly washed in basic uptake buffer and subsequently placed in basic uptake buffer containing 0.5 mM Mg^2+^ (containing ±10% ^25^Mg^2+^) for 5 minutes. After washing three times with ice-cold PBS, the cells were lysed in nitric acid and subjected to ICP-MS analysis.

### Morpholino knockdown and rescue experiments

Wild-type Tupfel long-fin zebrafish were bred and raised under standard conditions (28.5°C and 14 h of light: 10 h of dark cycle) in accordance with international and institutional guidelines. Zebrafish eggs were obtained from natural spawning. The following antisense oligonucleotides (MOs) were raised against the translational start site of *cnnm2a* and *cnnm2b*, along with the standard mismatch control MO (Gene Tools, Philomath, OR, USA): *cnnm2a*, 5′-GCGGTCCATTGCTCTGCCATGTTGA-3′; *cnnm2b*, 5′-ACCGACGGTTCTGCCATGTTGATAA-3′; and the negative control (standard mismatch MO), directed against a human β-globin intron mutation, 5′-CCTCTTACCTCAGTTACAATTTATA. The underlined areas indicate the complementary sequences to the initial methionines of *cnnm2a* and *cnnm2b*. MOs were diluted in deionized, sterile water supplemented with 0.5% (w/v) phenol red and injected in a volume of 1 nl into the yolk of one- to two-cell stage embryos using a Pneumatic PicoPump pv280 (World Precision Instruments, Sarasota, FL, USA). Wild-type (uninjected) embryos were also included in the experiments to control for the effects of the injection procedure *per se*. To determine the most effective dose of the *cnnm2a-* and *cnnm2b-*MO, 2, 4 and 8 ng were injected. In these experiments, control embryos were injected with 8 ng of the standard mismatch control MO (the highest dose). After injection, embryos from the same experimental condition were placed in 3 Petri dishes (at a maximum density of 45 embryos/dish, allowing statistical comparisons between survivals in the different experimental conditions) and cultured at 28.5°C in E3 embryo medium (5 mM NaCl, 0.17 mM KCl, 0.33 mM CaCl_2_, 0.33 mM MgSO_4_), which was refreshed daily. As criteria for subsequent experiments, the dose of MO that caused major effects and induced a percentage of mortality non-significantly different from controls was injected (2 ng for *cnnm2a*-MO and 8 ng for *cnnm2b*-MO). In experiments that implied exposure to high Mg^2+^ concentrations, the high Mg^2+^ medium had a composition of 5 mM NaCl, 0.17 mM KCl, 0.33 mM CaCl_2_ and 25 mM MgSO_4_. In order to control for the specificity of the MOs blocking the translation of *cnnm2a* and *cnnm2b*, as well as for toxic off-target effects, *in vivo* cRNA rescue experiments were performed [40]. For these experiments, mouse wild-type CNNM2 and mutant (p.Glu357Lys) CNNM2 cRNAs were prepared using the mMESSAGE mMACHINE Kit (Ambion, Austin, TX, USA) according to the manufacturer's instructions. The cRNAs, in an amount of 50 pg, as based on other studies [26], were (co)injected together with MOs as described above. Zebrafish embryos and larvae were phenotyped at 25 hpf or 5 dpf, respectively.

### Analysis of phenotypes in embryos and larvae

For the analyses of brain phenotypes, the brain rudiment of zebrafish embryos at 25 hpf was observed for morphological changes under a Leica MZFLIII microscope (Leica Microsystems Ltd, Heerburgg, Germany). Morphological phenotypes, which also included the brain, were also analysed in larvae at 5 dpf. Embryos or larvae were classified into different classes of phenotypes on the basis of comparisons with stage-matched control embryos of the same clutch. In *cnnm2a* morphant embryos (25 hpf), two phenotype classes were distinguished: class I, normal; and class II, embryos with underdeveloped MHB. In *cnnm2a* morphant larvae (5 dpf), the following classes were distinguished: class I, normal; class II, normal with non-inflated swim bladder; class III, larvae with enlarged pericardial cavity (edema) and non-inflated swim bladder; class IV, larvae with notochord defects, enlarged pericardial cavity and non-inflated swim bladder; and class V, larvae with severely enlarged pericardial cavity, notochord defects and non-inflated swim bladder. In *cnnm2b* morphant embryos (25 hpf), three different phenotypes were distinguished: class I, normal; class II, embryos with underdeveloped MHB; and class III, embryos with underdeveloped MHB and enlarged tectum. In *cnnm2b* morphant larvae (5 dpf), the number of different phenotypes distinguished were four: class I, normal; class II, normal with non-inflated swim bladder; class III, larvae with enlarged pericardial cavity (edema) and non-inflated swim bladder; and class IV, larvae with widened outer cerebrospinal fluid spaces, kidney cysts, severely enlarged pericardial cavity and non-inflated swim bladder. Representative images were obtained with a DFC450C camera (Leica Microsystems Ltd) after anaesthetising embryos or larvae with tricaine/Tris pH 7.0 solution. Prior to anaesthesia and image acquisition, zebrafish embryos were manually dechorionated.

### Magnesium determinations in embryos and larvae

Zebrafish embryos or larvae were anesthetized with tricaine/Tris pH 7.0 solution and 5–7 individuals were pooled as one sample. Samples were then snap frozen in liquid nitrogen and stored at −80°C in order to ensure euthanasia of animals and remained at these storage conditions until the beginning of the analytical procedures.

Analytical procedures started by quickly washing the samples with nanopure water in order to avoid contamination of remaining waterborne Mg^2+^. The washing procedure was repeated twice. Fish were then dried at 65°C for 1.5 hours, at which time 2.5 µl of HNO_3_ (≥65%, Sigma) was added to each tube. Samples were digested at 65°C during 1.5 hours. After, digested samples were diluted 1∶10 with 22.5 µl nanopure water. The total Mg content in each sample was determined with a commercial colorimetric assay (Roche Diagnostics, Woerden, The Netherlands) following the manufacturer's protocol. Blanks (HNO_3_ diluted 1∶10 with nanopure water) were added during assays and values were equal to zero. Within-run precision and accuracy was controlled by means of an internal control Precinorm (Roche Diagnostics). Samples from embryos exposed to 25 mM Mg^2+^ were further diluted (1∶20). Furthermore, samples were normalized by protein content, which was determined in 1∶20 (embryos at 25 hpf) or 1∶50 (larvae at 5 dpf) diluted samples using the Pierce BCA protein assay kit (Pierce Biotechnology, Rockford, IL, USA).

### Total RNA isolation, cDNA synthesis and quantitative real-time PCR analysis

Zebrafish embryos or larvae at specific developmental times (6, 12, 24, 48, 72, 96 and 120 hpf) were anaesthetised with tricaine/Tris pH 7 solution and 10 individuals were pooled as one sample. RNA isolation, cDNA synthesis and quantitative real-time PCR (RT-qPCR) measurements were carried out as previously described using validated *cnnm2a* and *cnnm2b* primers [18]. Samples were normalized to the expression level of the housekeeping gene *elongation factor-1α* (*elf1α*) [18]. Relative mRNA expression was analysed using the Livak method (2^−ΔΔCt^), where results are expressed relative to the gene expression at 6 hpf (time point chosen as calibrator).

### Spontaneous contraction analysis and touch-evoked escape behaviour

At 25 hpf, 10 zebrafish embryos per Petri dish (n = 30 per experimental condition) were randomly selected. The number of complete body contractions each zebrafish made in 30 second period was counted and was used as indicative of motor neuron activity [27]. Representative videos of each experimental condition were taken using Leica Application Suite (Leica Microsystems Ltd) and a Leica MZFLIII microscope (Leica Microsystems Ltd) equipped with a DFC450C camera (Leica Microsystems Ltd).

For the analysis of the touch-evoked escape behaviour, 10 zebrafish larvae per Petri dish (n = 30 per experimental condition) were randomly selected. Touch-evoked escape behaviours were elicited by touching a larva in the tail up to 6 times with a pair of forceps at 5 dpf. Three categories were distinguished, responders, late responders and non-responders, to which the following scores were given: 3 points for responders: fish quickly react (swimming or flicking the tail) to the stimuli after 1 or 2 twitches; 2 points for late responders: fish react (swimming or flicking the tail) to the stimuli after 3, 4 or 5 twitches; and 1 point for non-responders: fish do not react to the stimuli after more than 5 twitches. Representative videos were recorded with the system described above.

### Statistical analysis

All results are depicted as mean ± standard error of the mean (SEM). Statistical analyses were conducted by one- or two-way (for experiments where zebrafish embryos were exposed to different Mg^2+^ concentrations, then two factors of variance appear: Mg^2+^ concentration and treatment) ANOVA. Where appropriate, data were logarithmically transformed to fulfil the requirements for ANOVA, but all data are shown in their decimal values for clarity. When data did not comply with the premises of the parametric ANOVA, data were analyzed using a Kruskal-Wallis ANOVA on ranks. Tukey's post-test was used to identify significantly different groups. Statistical significance was accepted at *P*<0.05.

## Supporting Information

Figure S1 Impairment of touch-evoked escape behaviour in *cnnm2a* morphant zebrafish. Touch-evoked escape behaviour score in zebrafish *cnnm2a* morphants at 5 dpf after injection of 2 ng control-MO/embryo, 2 ng *cnnm2a*-MO/embryo, 2 ng *cnnm2a*-MO/embryo+50 pg wild-type (WT) CNNM2 cRNA/embryo, or 2 ng *cnnm2a*-MO/embryo+50 pg mutant (MT, p.Glu357Lys) CNNM2 cRNA/embryo. Three categories were distinguished, responders, late responders and non-responders, to which the following scores were given: 3 points for responders: fish quickly react (swimming or flicking the tail) to the stimuli after 1 or 2 twitches; 2 points for late responders: fish react (swimming or flicking the tail) to the stimuli after 3, 4 or 5 twitches; and 1 point for non-responders: fish do not react to the stimuli after more than 5 twitches. The upper part of the figure shows frames of videos showing touch-evoked escape contractions at 5 dpf of control and morphant zebrafish larvae. Time of each video frame is indicated in centisenconds (cs). Data are shown as mean ± SEM (n = 30). Different letters indicate significant differences between mean values in experimental groups (*P*<0.05).
(TIF)

Figure S2 Impairment of touch-evoked escape behaviour in *cnnm2b* morphant zebrafish. Touch-evoked escape behaviour score in zebrafish *cnnm2b* morphants at 5 dpf after injection of 8 ng control-MO/embryo, 8 ng *cnnm2b*-MO/embryo, 8 ng *cnnm2b*-MO/embryo+50 pg wild-type (WT) CNNM2 cRNA/embryo, or 2 ng *cnnm2b*-MO/embryo+50 pg mutant (MT, p.Glu357Lys) CNNM2 cRNA/embryo. Three categories were distinguished, responders, late responders and non-responders, to which the following scores were given: 3 points for responders: fish quickly react (swimming or flicking the tail) to the stimuli after 1 or 2 twitches; 2 points for late responders: fish react (swimming or flicking the tail) to the stimuli after 3, 4 or 5 twitches; and 1 point for non-responders: fish do not react to the stimuli after more than 5 twitches. The upper part of the figure shows frames of videos showing touch-evoked escape contractions at 5 dpf of control and morphant zebrafish larvae. Time of each video frame is indicated in centisenconds (cs). Data are shown as mean ± SEM (n = 30). Different letters indicate significant differences between mean values in experimental groups (*P*<0.05).
(TIF)

Movie S1 Spontaneous contraction frequency in zebrafish embryos (25 hpf) injected with a dose of 2 ng control-MO/embryo.
(AVI)

Movie S2 Spontaneous contraction frequency in zebrafish embryos (25 hpf) injected with a dose of 2 ng *cnnm2a*-MO/embryo.
(AVI)

Movie S3 Spontaneous contraction frequency in zebrafish embryos (25 hpf) co-injected with a dose of 2 ng *cnnm2a*-MO/embryo and 50 pg of mutant (p.Glu357Lys) CNNM2 cRNA.
(AVI)

Movie S4 Spontaneous contraction frequency in zebrafish embryos (25 hpf) injected with a dose of 8 ng control-MO/embryo.
(AVI)

Movie S5 Spontaneous contraction frequency in zebrafish embryos (25 hpf) injected with a dose of 8 ng *cnnm2b*-MO/embryo.
(AVI)

Movie S6 Spontaneous contraction frequency in zebrafish embryos (25 hpf) co-injected with a dose of 8 ng *cnnm2b*-MO/embryo and 50 pg of mutant (p.Glu357Lys) CNNM2 cRNA.
(AVI)

Table S1 Primer sequences for mutagenesis PCR.
(DOCX)
