# Supplementary figures and images for: CNNM2 Mutations Cause Impaired Brain Development and Seizures in Patients with Hypomagnesemia
Source: PLoS Genet. 2014 Apr 3;10(4):e1004267. doi: 10.1371/journal.pgen.1004267 (PMC3974678; doi:10.1371/journal.pgen.1004267)

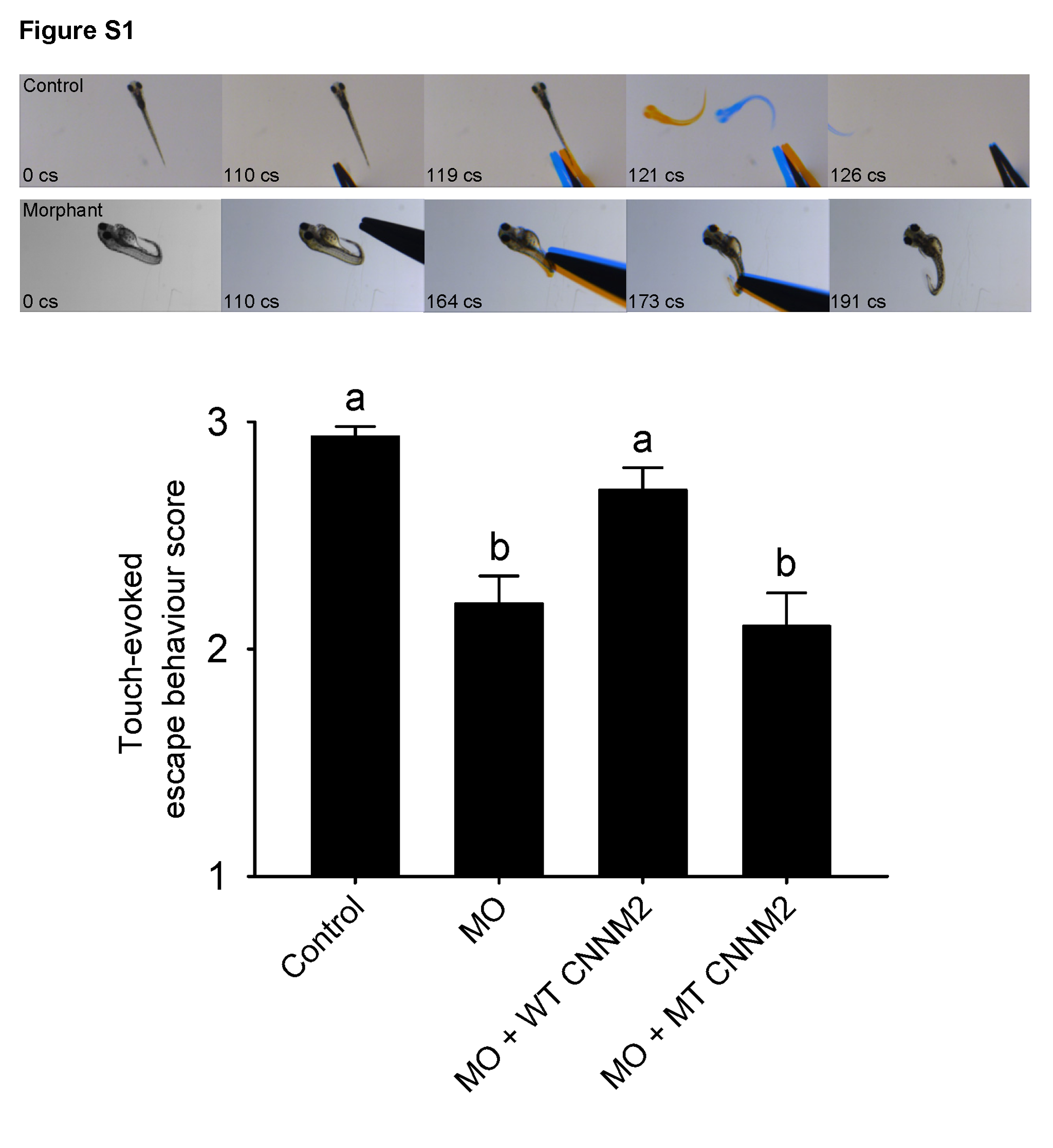

Supplement: Figure S1 — Impairment of touch-evoked escape behaviour in cnnm2a morphant zebrafish. Touch-evoked escape behaviour score in zebrafish cnnm2a morphants at 5 dpf after injection of 2 ng control-MO/embryo, 2 ng cnnm2a-MO/embryo, 2 ng cnnm2a-MO/embryo+50 pg wild-type (WT) CNNM2 cRNA/embryo, or 2 ng cnnm2a-MO/embryo+50 pg mutant (MT, p.Glu357Lys) CNNM2 cRNA/embryo. Three categories were distinguished, responders, late responders and non-responders, to which the following scores were given: 3 points for responders: fish quickly react (swimming or flicking the tail) to the stimuli after 1 or 2 twitches; 2 points for late responders: fish react (swimming or flicking the tail) to the stimuli after 3, 4 or 5 twitches; and 1 point for non-responders: fish do not react to the stimuli after more than 5 twitches. The upper part of the figure shows frames of videos showing touch-evoked escape contractions at 5 dpf of control and morphant zebrafish larvae. Time of each video frame is indicated in centisenconds (cs). Data are shown as mean ± SEM (n = 30). Different letters indicate significant differences between mean values in experimental groups (P<0.05). (TIF) [file pgen.1004267.s001.tif]

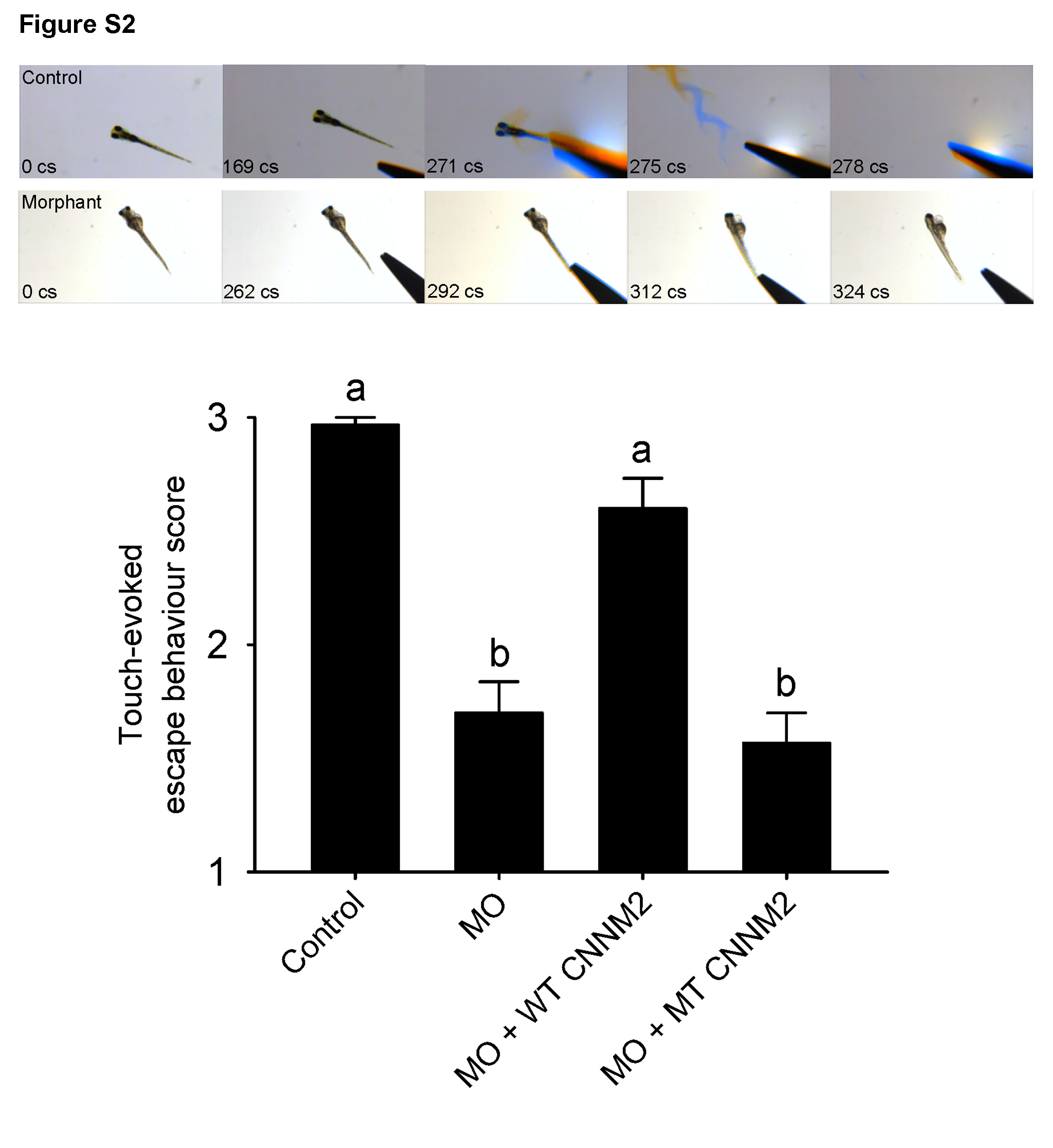

Supplement: Figure S2 — Impairment of touch-evoked escape behaviour in cnnm2b morphant zebrafish. Touch-evoked escape behaviour score in zebrafish cnnm2b morphants at 5 dpf after injection of 8 ng control-MO/embryo, 8 ng cnnm2b-MO/embryo, 8 ng cnnm2b-MO/embryo+50 pg wild-type (WT) CNNM2 cRNA/embryo, or 2 ng cnnm2b-MO/embryo+50 pg mutant (MT, p.Glu357Lys) CNNM2 cRNA/embryo. Three categories were distinguished, responders, late responders and non-responders, to which the following scores were given: 3 points for responders: fish quickly react (swimming or flicking the tail) to the stimuli after 1 or 2 twitches; 2 points for late responders: fish react (swimming or flicking the tail) to the stimuli after 3, 4 or 5 twitches; and 1 point for non-responders: fish do not react to the stimuli after more than 5 twitches. The upper part of the figure shows frames of videos showing touch-evoked escape contractions at 5 dpf of control and morphant zebrafish larvae. Time of each video frame is indicated in centisenconds (cs). Data are shown as mean ± SEM (n = 30). Different letters indicate significant differences between mean values in experimental groups (P<0.05). (TIF) [file pgen.1004267.s002.tif]
