# Supplementary material for: CNNM2 Mutations Cause Impaired Brain Development and Seizures in Patients with Hypomagnesemia
Source: PLoS Genet. 2014 Apr 3;10(4):e1004267. doi: 10.1371/journal.pgen.1004267 (PMC3974678; doi:10.1371/journal.pgen.1004267)
Supplement: Table S1 — Primer sequences for mutagenesis PCR. (DOCX) [file pgen.1004267.s009.docx]

**Table S1. Primer sequences for mutagenesis PCR**

| **Mutation** | **Sequence** |  |
| --- | --- | --- |
| E122K | GGTCCCGCATCGCCTTCACTAAGCACGAGCGGCGCCGGCAC | F |
|  | GTGCCGGCGCCGCTCGTGCTTAGTGAAGGCGATGCGGGACC | R |
| S269W | CATCTCGCTGCTGCTGTGCCTGTGGGGCATGTTCAGCGGCCTCAAC | F |
|  | GTTGAGGCCGCTGAACATGCCCCACAGGCACAGCAGCAGCGAGATG | R |
| L330F | GTACTGGTCAACACCACGTTCACCATCCTGCTGGACGAC | F |
|  | GTCGTCCAGCAGGATGGTGAACGTGGTGTTGACCAGTAC | R |
| E357K | GGCATCGTCATCTTCGGAAAAATCGTGCCCCAAGCCATC | F |
|  | GATGGCTTGGGGCACGATTTTTCCGAAGATGACGATGCC | R |
